# Disruption of Circadian Rhythms by Ambient Light during Neurodevelopment Leads to Autistic-like Molecular and Behavioral Alterations in Adult Mice

**DOI:** 10.3390/cells10123314

**Published:** 2021-11-26

**Authors:** Kun Fang, Dong Liu, Salil S. Pathak, Bowen Yang, Jin Li, Ramanujam Karthikeyan, Owen Y. Chao, Yi-Mei Yang, Victor X. Jin, Ruifeng Cao

**Affiliations:** 1 Department of Molecular Medicine, University of Texas Health San Antonio, San Antonio, TX 78229, USA; FangK@livemail.uthscsa.edu (K.F.); yangb@livemail.uthscsa.edu (B.Y.); 2 Department of Biomedical Sciences, University of Minnesota Medical School, Duluth, MN 55812, USA; cyyzld@gmail.com (D.L.); spathak@d.umn.edu (S.S.P.); lijin0213@126.com (J.L.); karthikbiology@gmail.com (R.K.); ychao@d.umn.edu (O.Y.C.); 3 Department of Neuroscience, University of Minnesota Medical School, Minneapolis, MN 55455, USA

**Keywords:** circadian rhythm, neurodevelopmental disorder, translational control, mTOR, MAPK, autism

## Abstract

Although circadian rhythms are thought to be essential for maintaining body health, the effects of chronic circadian disruption during neurodevelopment remain elusive. Here, using the “Short Day” (SD) mouse model, in which an 8 h/8 h light/dark (LD) cycle was applied from embryonic day 1 to postnatal day 42, we investigated the molecular and behavioral changes after circadian disruption in mice. Adult SD mice fully entrained to the 8 h/8 h LD cycle, and the circadian oscillations of the clock proteins, PERIOD1 and PERIOD2, were disrupted in the suprachiasmatic nucleus and the hippocampus of these mice. By RNA-seq widespread changes were identified in the hippocampal transcriptome, which are functionally associated with neurodevelopment, translational control, and autism. By western blotting and immunostaining hyperactivation of the mTOR and MAPK signaling pathways and enhanced global protein synthesis were found in the hippocampi of SD mice. Electrophysiological recording uncovered enhanced excitatory, but attenuated inhibitory, synaptic transmission in the hippocampal CA1 pyramidal neurons. These functional changes at synapses were corroborated by the immature morphology of the dendritic spines in these neurons. Lastly, autistic-like animal behavioral changes, including impaired social interaction and communication, increased repetitive behaviors, and impaired novel object recognition and location memory, were found in SD mice. Together, these results demonstrate molecular, cellular, and behavioral changes in SD mice, all of which resemble autistic-like phenotypes caused by circadian rhythm disruption. The findings highlight a critical role for circadian rhythms in neurodevelopment.

## 1. Introduction

The circadian clock endogenously drives approximately 24-h rhythms in all animals. Circadian rhythms are fundamentally important in regulating gene expression, neurophysiology, and animal behavior across the lifespan [1]. During brain development, the circadian clock regulates neurogenesis, migration, and progenitor cell differentiation. In the adult brain, circadian rhythms regulate neuronal excitability, synaptic plasticity, learning and memory, mood, and social behaviors [2]. The cellular circadian clock is composed of interlocking transcriptional–translational feedback loops. Decades of research has identified about a dozen clock genes that are ubiquitously expressed in all mammalian cells [3]. The expression of clock genes in different systems is orchestrated and synchronized by the master circadian pacemaker, the suprachiasmatic nucleus (SCN) in the hypothalamus. Ambient light regulates rhythms in the SCN and synchronizes the endogenous body rhythms with the environmental LD cycles. It is generally thought that circadian rhythms are essential for maintaining body health. Aberrant light exposure, such as light at night, shift work, or transmeridian travel can disrupt circadian rhythms and cause their desynchronization in different systems of the body. In the short term, circadian desynchronization can cause “jet lag” symptoms and sleep disorders [4]. The long-term consequences of circadian disruption during animal development remain elusive. The molecular and cellular changes in the adult brain after chronic circadian disruption during neurodevelopment have never been investigated. 

Autism spectrum disorders (ASDs) are a group of neurodevelopmental disorders characterized by impaired social interaction and communication, and restricted, repetitive, or stereotyped movements (DSM-V). ASD affect 1 in 54 children in the U.S. (CDC). To date, no preventive or therapeutic strategies have demonstrated consistent benefits in treating the core symptoms of ASD. There is, therefore, an urgent need to uncover new pathogenic mechanisms of ASD from experimental studies. Emerging clinical evidence indicates that dysfunctions of the circadian clock may be involved in ASD pathogenesis [5]. For example, clock gene polymorphisms are frequently found in ASD. Single-nucleotide polymorphisms and de novo loss-of-function variants of multiple clock genes are identified in ASD, including *Per1*, *Per2*, *Npas2*, *Nr1d1*, *Rorα*/*β*, and *CkIε*, which indicates that the functional abnormality of these clock genes may be involved in ASD [6,7,8,9,10]. In addition, sleep problems and abnormal levels of circadian hormones are common comorbidities in ASD individuals. It is estimated that 50~80% of ASD children have sleep problems, compared to less than 30% in the general children population [11]. Abnormal temporal profiles of cortisol and melatonin, which are indicators of the circadian clock function, are frequently found in ASD [12,13]. Together, these findings indicate underlying impairments of the body clock function in ASD. 

Despite these correlation studies, however, no experimental evidence has yet established a causal link between circadian rhythm disruption and ASD. Filling this gap may help us to better understand the mechanisms of the circadian regulation of neurodevelopment and to develop novel strategies to treat circadian sleep problems, which are frequently found in neurodevelopmental disorders. Here, to investigate the effects of circadian disruption during neurodevelopment, we established a “Short Day” (SD) mouse model, in which the circadian rhythms in whole animals were chronically disrupted by ambient light throughout the period of embryonic and postnatal development, from embryonic day 1 to postnatal day 42 (E1-P42). We investigated the molecular and behavioral changes in adult SD mice as compared to the control mice that were constantly maintained under the standard 12 h/12 h light/dark cycle. Interestingly, we identified a combination of molecular, cellular, and behavioral changes in the SD mice, all of which resemble autism-like phenotypes in these mice. Our findings highlight a critical role for circadian rhythms in neurodevelopment and provide experimental evidence that links the environmental disruption of circadian rhythms to the pathogenesis of ASD.

## 2. Materials and Methods

### 2.1. Animals

**Figure 1:**
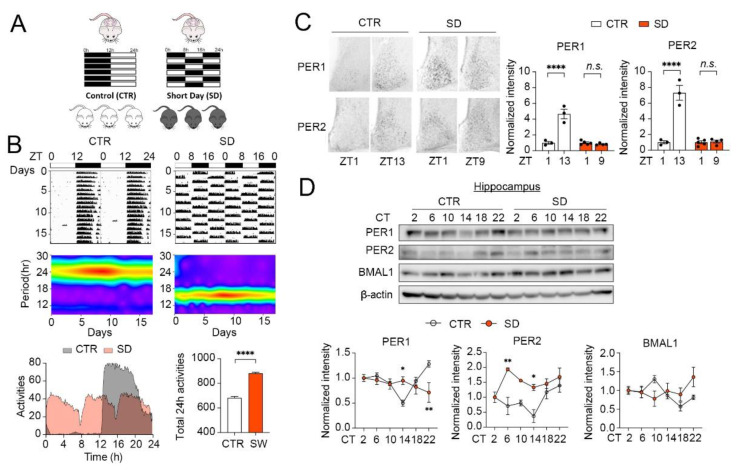
Daily rhythms of animal locomotor activities and clock gene expression are disrupted in SD mice. (**A**) A diagram indicating the strategy to generate the SD mice. (**B**) Top: Representative actograms of mouse wheel-running activities in control (CTR) and Short Day (SD) mice. Middle: Morlet wavelet transform analysis below indicates that CTR mice entrained to the 12 h/12 h light/dark cycle and exhibited a circadian period of 24 h. In contrast, SD mice entrained to the 8 h/8 h light/dark cycle and exhibited a circadian period of 8 h. Bottom: Average activities of CTR (n = 4) and SD (n = 3) mice across 24 h. Note that the SD mice exhibited a higher level of total activities in 24 h as compared with the CTR mice. (**C**) Representative microscopic images of immunostaining for PER1 and PER2 in the SCN. ZT1: one hour after light-on for CTR and SD mice; ZT13 and ZT9: 1 h after light-off for CTR and SD mice. Quantitative analysis of staining intensity is shown to the right. The levels at ZT13 and ZT9 were normalized according to the levels at ZT1 in the CTR and the SD mice, respectively. Note that PER1 and Per2 levels were not different between ZT1 and ZT9 in SD mice. Data are displayed as individual values and mean ± SEM. n = 3 in CTR and n = 4–5 in SD. **** *p* < 0.0001; n.s., not significant vs. ZT1. (**D**) Western blotting images indicate that PER1, PER2, and BMAL1 exhibited circadian oscillations in the hippocampi of the CTR, but not the SD, mice. Quantitation of protein levels is shown below. For this experiment mice were kept in constant darkness for 48 h and hippocampal samples were harvested every 4 h in the next 24 h. Data are displayed as mean ± SEM. n = 3 in CTR and SD. ** *p* < 0.01; * *p* < 0.05 vs. CTR.

C57BL/6J breeder mice were purchased from the Jackson Laboratory (Stock No: 000664; RRID: IMSR_JAX:000664). Animals were housed in the animal facility at the University of Minnesota, Duluth, with ad libitum access to mouse chow (LabDiet 5053) and tap water. The room temperature was maintained at 22 ± 1 °C, and the humidity was at 35–45%. Mouse colonies were maintained in the standard 12 h/12 h light/dark (LD) cycle. To create SD mice, six-week-old male and female breeders were transferred to the 8 h/8 h LD cycle (100 lux). Breeding pairs were set up and maintained in the 8 h/8 h LD cycle throughout gestation. Pups were also kept in the 8 h/8 h LD cycle until they became 42 days old (Figure 1A). Control (CTR) mice were kept and bred in the 12 h/12 h LD cycle. All procedures were approved by the Institutional Animal Care and Use Committee at the University of Minnesota.

### 2.2. Circadian Behavioral Assay

Six-week-old SD and CTR mice were transferred and individually housed in cages equipped with running wheels, and kept in either SD or CTR light cycles for 17 d. The wheel-running activities were recorded and analyzed by ClockLab software (Actimetrics, Wilmette, IL, USA).

### 2.3. Brain Tissue Processing, Immunostaining, and Microscopic Imaging Analysis

Under the indicated conditions, the mice were sacrificed by cervical dislocation and decapitation. The brains were rapidly harvested. Brain tissues were processed and cut into 40-µM sections, as previously reported. Immunohistochemistry was performed as published in [14], using one of the following primary antibodies: rabbit anti-phospho-eIF4E (Ser209) antibody (1:1000 final dilution; Novus Biologicals, NBP1-19923; RRID: AB_1641951); rabbit anti-phospho-S6 (Ser240/244) antibody (1:1000 final dilution; Cell Signaling Tech, 2215); rabbit anti-PER1 antibody (1:3000, Millipore, AB2201); or goat anti-PER2 antibody (1:300, Santa Cruz, sc-7728). Next, sections were incubated for 1.5 h at room temperature in biotinylated anti-rabbit/goat IgG (1:400; Vector Laboratories, Burlingame, CA; RRID: AB_2687893), and then placed in an avidin/biotin HRP complex for 1 h (prepared according to the instructions of the manufacturer; Vector Laboratories; RRID: AB_2336819). Sections were washed three times in PBS for 10 min per wash between each labeling step. The signal was visualized using nickel-intensified DAB substrate (Vector Laboratories; RRID: AB_2336382) and sections were mounted on slides with Permount media (SP15-500, Fisher Scientific, Houston, TX, USA).

Bright-field microscopic images were captured using a digital camera mounted on an inverted DMi8 Leica microscope (Wetzlar, Germany). All photomicrographic datasets were statistically analyzed using Adobe Photoshop software (Adobe Systems Incorporated, San Jose, CA, USA), as previously described [15]. Briefly, for the PER1 and PER2 intensity analyses, 10× bright-field SCN images were digitally outlined, subtracted from the intensity of the adjacent lateral hypothalamus, and the normalized mean intensity values were determined after being normalized to the values in the control group. For the p-S6 and p-eIF4E intensity analyses, 1.2× sagittal brain images were used. Three squares of 10 × 10 pixels were used to outline the CA1, CA3, and DG regions of the hippocampus and the average intensity in each square was determined. After subtracting it from the intensity of a cortex region with no labeling, the normalized intensity was determined.

### 2.4. Protein Extraction and Western Blotting Analysis

The mice were sacrificed under the indicated conditions. The brains were dissected, snap-frozen in dry ice, and stored in −80 °C until protein extraction. The proteins were extracted, and Western blotting was done, as previously reported in [14]. Briefly, forebrain tissues were homogenized in ice-cold lysis buffer (20 mM HEPES pH 7.5, 100 mM NaCl, 0.05% Triton X-100, 1 mM DTT, 5 mM Na-beta-glycerophosphate, 0.5 mM Na-vanadate, 1 mM EDTA, protease inhibitors). The supernatant was obtained, and the protein concentration was quantified by Bradford assay. Protein lysates, in amounts of 50 µg, were resolved on a 10% SDS PAGE gel and transferred to a PVDF membrane (Bio-Rad). Membranes were blocked by 5% nonfat dry milk and incubated with one of the following primary antibodies overnight at 4 °C: anti-phospho-eIF4E (Ser209) antibody (1:1000; Novus Biologicals, NBP1-19923; RRID: AB_1641951); anti-eIF4E antibody (1:1000, BD Transduction Laboratories, 610270; RRID: AB_397665); anti-phospho-S6 (Ser240/244) antibody (1:2000; Cell Signaling Tech, 2217; RRID: AB_2798616); anti-S6 antibody (1:2000; Cell Signaling Tech, 2215); anti-phospho-S6K1 (Thr389) antibody (1:1000, Cell Signaling Tech, 9206, AB_2285392); anti-S6K1 (1:2000, Cell Signaling Tech, 9202); anti-p-mTOR (Ser2448) (1:1000, Cell Signaling Tech, 2971); anti-mTOR (1:1000, Cell Signaling, 2972), anti-p-MEK 1/2 (217/221) (1:1000, Cell Signaling Tech, 9121); anti-MEK (1:1000, Cell Signaling, 9122); anti-p-ERK (Thr202/204) (1:1000, Cell Signaling Tech, 9106); or anti-ER (1:2000, Santa Cruz, SC-93). The next day, blots were washed with 1X PBST and incubated in PBST (with 5% skim milk) with an HRP-conjugated secondary antibody (1:5000, GE Healthcare, donkey anti-rabbit: NA931, AB_772210; donkey anti-mouse: NA934, AB_772206) for 1.5 h and washed again. To detect immunoreactivity, blots were incubated with enhanced chemiluminescent reagent (Perkin Elmer) and imaged on an Odyssey Fc Imaging System (LI-COR Biosciences). The relative intensities of the blots were quantified using Adobe Photoshop.

### 2.5. RNA-seq and Bioinformatic Analysis

The mice were sacrificed, and the hippocampal tissue was rapidly dissected and snap-frozen in dry ice and stored in −80 °C until RNA extraction. Total RNA was extracted by the ZYMO Research Quick-RNA MiniPrep kit from lysed hippocampal tissue in TRIzol Reagent (Thermosphere, 15596026), and then most of the gDNA was removed with the spin-away filter. After that, the mixture of RNA was transferred with ethanol to a Zymo-Spin IIICG column to remove trace DNA by DNase I on the column, then washed twice with RNA wash buffer, followed by elution with 50 μL DNase/RNase-free water. The RNA-seq library was prepared with the NEBNext^®^ Poly(A) mRNA Magnetic Isolation Module (NEB #E7490). The Oligo dT Beads were washed with RNA binding buffer and incubated with a total of 1 μg of RNA to purify the mRNA, followed with more washing by the bead-washing buffer. Then, the mRNA was eluted with elution buffer and reverse-transcribed. After that, the first and the second strand cDNA were synthesized. After the purification of the double-stranded DNA, adaptor was added. Adaptor-ligated DNA was enriched by PCR followed by purification, and then the DNA library was sequenced with Illumina HiSeq3000.

The quality control for the RNA-seq data was first performed by Trim Galore and was mapped to mm10 by Hisat2 with its default parameters [16]. The read counts for each gene were calculated by featureCounts [17], with the parameters, −s 2 and −M. Differentially expressed genes (DEGs) were identified by DESeq2 [18], with the cutoffs as abs (log2FoldChange) ≥ 0.58, and a *p*-value ≤ 0.05. A volcano plot and a heatmap of the DEGs were drawn with customed R script with the Pheatmap package [19]. The human homology genes were then mapped from mouse genes by a custom script. The protein–protein interaction networks were generated from STRING v11.5 [20].

ASD cohort data were acquired from the GEO database, with the GEO accession numbers: GSE28521, GSE102741, GSE64018, GSE51264, and GSE59288 [21,22,23,24]. The number of ASD/CTR samples in GSE102741 was balanced by randomly selecting control samples with a sample function in R. To identify the mice-cohort DEGs, we first performed linear regression *t*-tests with the equation:

(1) $$Expression=\alpha *Age+\beta *Offset_{ASD-CTR}+\gamma *Age:Offset_{ASD-CTR}\;$$

where $Age:Offset_{ASD-CTR}$ means the interaction between Age and $Offset_{ASD-CTR}$; and α,β,γ are the coefficients learned from the least square regression. Then, we checked if the coefficient of the interaction term, γ, was smaller than 0.1. If γ was less than the cutoff, 0.1, we hypothesized that the ASD and CTR groups shared the same slope and we then performed a simpler *t*-test model:

(2) $$Expression=\alpha *Age+\beta *Offset_{ASD-CTR}.$$

The mouse-cohort DEGs were finally identified on the basis of: 1. Linear regression *t*-test p value ≤0.05; and 2. FoldChange≥1.25, where $FoldChange=\frac{mean\left(expressions\;in\;the\;ASD\;samples\right)}{mean\left(expression\;in\;the\;CTR\;samples\right)}$. After obtaining the mouse-cohort DEGs for each cohort, we investigated the overlapping mouse-cohort DEGs between cohorts with a Venn diagram [25], and we used the learning vector quantization method in the Caret package to calculate the importance score for mice-cohort DEGs, in the data of Liu et al., on the basis of the annotated “ADI-R Class” in the original paper.

### 2.6. Electrophysiological Recording

Briefly, horizontal hippocampal slices were sectioned at a thickness of 300 μm, using a vibratome (VT 1200S, Leica) in ice-cold modified artificial cerebral spinal fluid (ACSF) containing (in mM): sucrose (217.6); KCl (3); glucose (10); NaH_2_PO_4_ (2.5); NaHCO_3_ (26); MgCl_2_ (2); and CaCl_2_ (2), and were continuously bubbled in 95% O_2_ and 5% CO_2_ (pH 7.4). Slices were incubated in oxygenated standard ACSF, including (in mM): NaCl (125); KCl (2.5); glucose (10); NaH_2_PO_4_ (1.25); sodium pyruvate (2); myo-inositol (3); ascorbic acid (0.5); NaHCO_3_ (26); MgCl_2_ (1); and CaCl_2_ (2) (pH 7.4), at 37 °C for 30 min prior to experimentation, and were then transferred to a recording chamber. Slices were continuously perfused with the standard ACSF, at a rate of approximately 1 mL/min, with supplements of NBQX (10 µM) and APV (50 µM) or bicuculline (10 µM) to block excitatory or inhibitory inputs, respectively. Miniature EPSCs and IPSCs from CA1 pyramidal neurons were recorded in the whole-cell voltage-clamp mode at −60 mV. Tetrodotoxin (TTX, 1 μM) was added to eliminate spontaneous spike firing. To record the EPSCs, the intracellular solution contained (in mM): K-gluconate (97.5); CsCl (32.5); EGTA (5); HEPES (10); MgCl_2_ (1); TEA (30); and lidocaine *N*-ethyl bromide (3) (pH 7.3). To record the IPSCs, the intracellular solution contained (in mM): K-gluconate (50); CsCl (80); EGTA (5); HEPES (10); MgCl_2_ (1); TEA (30); and lidocaine N-ethyl bromide (3) (pH 7.3). The electrophysiological recordings were acquired online, filtered at 4 kHz, and digitized at 50 kHz with a dual-channel amplifier, (MultiClamp 700 A, Molecular Devices) and digitizer (Digidata 1322 A, Molecular Devices). Data were analyzed offline with pClamp 10 (Molecular Devices), MiniAnalysis (Synaptosoft), and Excel 2016 (Microsoft).

### 2.7. Analysis of Neuronal Morphology by Golgi–Cox Staining

The Golgi–Cox staining of the mouse brains was performed using the Rapid Golgi Kit (FD NeuroTechnologies, MD, USA), according to the manufacturer’s instructions. Briefly, whole brains were isolated from each animal, rinsed once in Milli-Q water, quickly immersed into impregnation solution (A + B), and were then stored at room temperature in the dark for three weeks. Sections of a 100-μm size were cut, processed, and mounted, following the protocol provided with the kit. Hippocampal sections were imaged on a Nikon light microscope with a 100× oil immersion lens. To measure the spine densities on the apical shaft dendrites from the hippocampal CA1 area, the number of spines on each successive 25-mm segment was counted, starting at the soma and continuing to the end of the dendrite. Densities for each segment and for each neuron were pooled to get an average spine density per animal. For each neuron, the spine morphology was determined for the first 10 spines in every 25-μm bin along the apical shaft. Spines were assigned one of the following five morphological categories: A: Thin; B: Stubby; C: Mushroom; D: Filopodia; and E: Branched. B and C are classified as "mature spines” and A, D and E are classified as “immature spines”, as published in [26,27]. The percentages of the mature and immature spines were determined.

### 2.8. Puromycin Treatment and De Novo Protein Synthesis Analysis

A puromycin-labeling assay was used to measure the de novo protein synthesis [28]. The mice were injected with puromycin (125 mg/kg body weight) intraperitoneally and sacrificed 60 min later. The hippocampal tissue was collected, proteins were extracted, as described earlier, and Western blotting was done using the anti-puromycin antibody (1:1000, MilliporeSigma, MABE343, AB_2566826). The protein synthesis level was determined by measuring the total lane signal from 15–250 kD and subtracting it from the intensity of the unlabeled lane signal. After being normalized to loading control, the normalized protein synthesis was presented as a percentage change relative to the control.

### 2.9. Mouse Behavioral Tests

Six- to eight-week-old CTR and SD mice were used in all behavioral tests, except for the ultrasonic vocalization (USV) test, in which 3~11-day-old pups were used. The male-to-female ratios were roughly 1:1 in all of the experiments. Unless otherwise indicated, all behavioral tests were performed under a dim red light (~20 lux at cage level) beginning at 2 h after light-off, when the mice were naturally active. Whenever applicable, the experiments were videorecorded by a high-resolution camera, and the videos were analyzed by the animal behavioral tracking software, ANY-maze (Stoelting Co., Wood Dale, IL, USA). The researchers were blinded to the mouse genotypes during analysis.

Three-chamber test. The three-chamber test was performed, as published in [29], using a clear polyvinyl chloride (PVC) apparatus (60 cm × 40 cm × 20 cm) with three compartments (20 cm × 40 cm × 20 cm). For habituation, a mouse was placed in the central compartment and was allowed to freely explore all three compartments for 10 min. After habituation, a stranger WT C57BL/6J mouse (stranger 1) of the same sex was caged inside a wire cup in one of the side compartments. An identical empty wire cup was placed in the other side of the compartment. The test mouse was allowed to freely explore all three compartments for 5 min. Next, a novel stranger WT C57BL/6J mouse (stranger 2) of the same sex was introduced into the empty wire cup. The test mouse was allowed to explore for another 5 min. Mouse movements and interactions were videorecorded, and the videos were analyzed using the ANY-maze video tracking system (Stoelting Co., Wood Dale, IL, USA) to determine the times and the number of entries to each chamber, as well as the time sniffing, which was defined as the test mouse placing its nose within 2 cm of the cup or placing its forelimbs on the cup.

Ultrasonic vocalization (USV) test. The USV test were performed at postnatal days 3, 7, and 11, as previously described in [30]. Pups were separated from the mother and placed individually in a soundproof chamber. Vocalizations from the pups were recorded for 5 min using an ultrasound microphone (M500-384, Pettersson, Sweden) connected to Batsound Touch Lite recording software (Pettersson Elektronik AB Uppsala Science Park, Uppsala, Sweden). The numbers of calls per min, and the call durations and frequencies were determined.

Olfactory habituation test. The test was performed in the standard polycarbonate mouse cage, as described in [29]. Briefly, mice were exposed to cotton-tipped swabs with different odors and the time spent sniffing each swab was recorded. A series of swabs with odors were used in the order of tap water, cinnamon extract (1:100, Watkins), butter (1:100, Watkins), and mouse cage swipes. These olfactory cues were designed to measure familiar and unfamiliar odors, with and without social valence. Each swab was presented for 3 min, and each odor was presented consecutively three times. Each test session was conducted in a new mouse cage containing fresh bedding. There was a 1-min interval between sessions. All sessions were videorecorded and analyzed to determine the time spent sniffing within 2 cm of the swab.

Marble burying test. The marble burying test was performed to assess repetitive behaviors and stereotypy, as published in [31]. Corncob bedding (5 cm in depth) was placed in a standard polycarbonate mouse cage and lightly tamped down to make a flat and even surface. Three rows of five marbles were gently placed 4-cm apart on the surface of the bedding. A mouse was gently placed into a corner of the cage and allowed to explore the cage freely for 30 min. After the session, the number of buried marbles was counted. A marble was counted as buried if it was at least 2/3 covered by the bedding.

Grooming. To assess repetitive behaviors and the stereotypy, mouse grooming was analyzed, as published in [32]. To record spontaneous grooming, the mice were habituated in a new standard polycarbonate mouse cage (38 cm × 22 cm × 16 cm) for 30 min, and then videorecorded for 10 min to analyze the self-grooming of all body regions during the recording time. To record the induced grooming, the mice were given a single water puff to mist the animal’s face and head and were then videorecorded for 5 min to analyze the self-grooming behaviors. The videos were inspected by blinded researchers and the number of grooming bouts and the durations of grooming were determined. Grooming was defined as either the stroke of the forepaws across the head, or face or body licking.

Novel object recognition (NOR) memory test. An NOR test was performed, as described in [33]. Briefly, 24 h before training, the mouse was first placed in an open field arena (40 cm × 40 cm × 30 cm), without objects for habituation, and was allowed to freely explore the arena for 10 min. On the training day, two identical objects (glass bottles) were placed at opposite corners (5 cm from the walls) of the arena. The mouse was then placed into the arena and was allowed to freely explore the objects for 10 min before returning to the home cage. At 24 h after training, one object was replaced by a novel object (a wooden cube). The mouse was returned to the arena to explore for 5 min. The time spent investigating novel or familiar objects, and the total distance traveled in the arena were determined. "Investigating" was defined as the mouse placing its nose within a 2-cm proximity to the object, or as touching the object with the nose or forepaws. The proportion of time spent investigating the novel or familiar object was calculated as Time _novel_ or Time _familiar_/Time _novel_ + Time _familiar_. The discrimination index (DI) was calculated as (Time _novel_ − Time _familiar_)/(Time _novel_ + Time _familiar_).

Object location memory (OLM) test. An OLM test was performed, as reported in [33]. At 24 h before training, the mouse was placed in an open field arena (40 cm × 40 cm × 30 cm), without objects for habituation, for 10 min. On the training day, the mouse was exposed to two identical objects (glass bottles), which were placed at two specific locations in the arena (as in the NOR test). The mouse was allowed to explore the arena and both objects for 10 min and was then returned to the home cage. After 24 h, one object was placed in the same position, while the other object was moved to a novel location (the adjacent corner, 5 cm from the walls). The mouse was returned to the arena to explore for 5 min and its behavior was videorecorded. The time spent investigating novel (N) or familiar (F) locations and the total distance traveled in the arena were determined. The proportion of time spent investigating objects at the novel or familiar locations was calculated as Time _novel_ or Time _familiar_ /(Time _novel_ + Time _familiar_). The discrimination index (DI) was calculated as (Time _novel_ − Time _familiar_)/(Time _novel_ + Time _familiar_).

### 2.10. Statistical Analysis

Statistical analysis was performed by SPSS software (SPSS Inc, Chicago, IL, USA). The quantified values were compared for statistics using the Student’s *t*-test (two groups) or the one-way ANOVA test (multiple groups). If there were mixed factors involved, two-way ANOVA were performed with regard to the confounding variations, followed by post hoc Bonferroni’s multiple comparisons. The values were presented as mean ± standard error of the mean (SEM), and *p* < 0.05 was considered to be of statistical significance.

## 3. Results

### 3.1. Daily Rhythms of Animal Locomotor Activity and Clock Gene Expression Are Disrupted in SD Mice

We first assessed whether the SD cycle was able to disrupt the diurnal rhythms of wheel-running activities in adult mice. Interestingly, the CTR mice were entrained to the standard 12 h/12 h LD cycles, whereas the SD mice were completely entrained to the 8 h/8 h LD cycle, which is an ultradian cycle that C57BL/6J mice normally cannot entrain to (Figure 1B). Indeed, the Morlet wavelet transform analysis revealed that the CTR mice exhibited a period length of 24 h in locomotor activities, and that the SD mice exhibited a period length of 16 h. As expected, the CTR mice exhibited consolidated activity bouts during the 12-h dark phase of the 24-h LD cycle. When averaged by multiple days, three bouts of activities were found in the SD mice within a 24-h period, with each bout spanned for about 8 h. As activities were restricted to the dark phase in the SD mice, the three bouts of the 8-h activities were caused by daily alternating the 8 h/8 h LD phases. As a result, the total locomotor activities in 24 h were increased in the SD mice compared with the CTR mice (Figure 1B, unpaired *t*-test, *p* < 0.0001). In LD cycles, mouse activity rhythms may be shaped by the masking effect of light. To determine whether molecular diurnal rhythms were also changed in the SD mice, we next detected the levels of the clock proteins, PER1 and PER2, in the SCN at 1 h after light-on (ZT1) and 1 h after light-off (ZT13 for CTR, ZT 9 for SD). Consistent with the published results, we found higher levels of PER1 and PER2 at ZT 13 compared with ZT 1 in the SCN of CTR mice, indicating the diurnal oscillations of the PER levels. Strikingly, the levels of PER1 and PER2 remained at similar levels at ZT1 and ZT 9 in the SD mice, demonstrating that the diurnal oscillations of clock proteins are disrupted in the SCN of SD mice (Figure 1C, two-way ANOVA, PER1: *p* < 0.0001; PER2: *p* < 0.0001).

As SCN is regulated by the ambient light/dark cycles and, in turn, synchronizes other brain regions, we next detected the PER1 and PER2 rhythms in an extra-SCN brain region, i.e., the hippocampus. Samples were collected every 4 h across a 24-h period, when the mice were kept in constant darkness. Consistent with the published findings, we found that the levels of PER1 and PER2 exhibited significant oscillations across 24 h in the hippocampus, with a peak detected at CT22-2 in the PER1 and PER2 levels, and at a trough at ~CT14 in the CTR mice. The level of BMAL1 was antiphase to the PER levels, with a peak at ~CT10 and a trough at CT18-22 in the hippocampi of the CTR mice. Interestingly, the diurnal profiles of PER1, PER2, and BMAL1 were disrupted in the hippocampi of the SD mice. No obvious peaks and troughs were detected in the PER1 and BMAL1 levels, and a peak was found at CT6 in the PER2 level in the SD mice (Figure 1D, two-way ANOVA, PER1: *p* = 0.0009; PER2: *p* = 0.0008; BMAL1: *p* = 0.036). These results indicate that the diurnal rhythms are disrupted at the behavioral and molecular levels in SD mice.

### 3.2. Genome-Wide Transcriptional Changes in the Hippocampi of SD Mice

**Figure 2:**
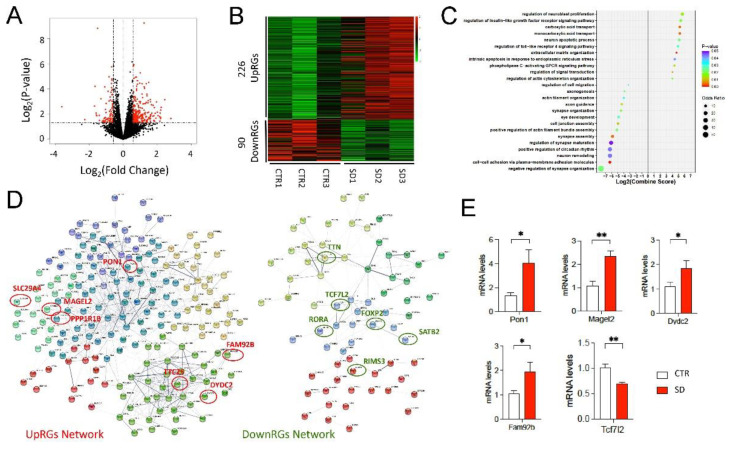
**Genome-wide transcriptional changes in the hippocampi of SD mice.** (**A**) Volcano plot shows the Log2 (fold-change) and Log2 (*p*-value) of the genes. DEGs are highlighted with red color. (**B**) Heatmap shows the Z-scored expression value of identified DEGs. We identified 226 UpRGs and 90 DownRGs in the SD mice. (**C**) Top 25 GO analysis results of DEGs. P-value is indicated by the color of points and the size of points is a proportion of the odds ratio. Negative log2 (combined score) indicates the pathway analyzed from the downregulated gene set. (**D**) DEGs network (protein–protein interaction from STRING v11.5), we found there were 7 upregulated and 6 downregulated genes that are included in the SFARI database. UpRGs are circled in red: slc29a4, magel2, fam92b, pon1, ttc25, dydc2, ppp1r1b; DownRGs are circled in green: satb2, rora, foxp2, rims3, ttn, tcf7l2. (**E**) Validation results of identified SFARI-overlapped DEGs by qRT-PCR. n = 3 in CTR and SD. ** *p* < 0.01; * *p* < 0.05 vs. CTR.

To identify the genome-wide transcriptional changes in the hippocampi of SD mice, we performed RNA-seq and identified a total of 316 DEGs, consisting of 226 upregulated genes (UpRGs) and 90 downregulated genes (DownRGs) (Figure 2A,B). We then used Enrichr [34] to perform the gene ontology (GO) functions (Figure 2C) and pathway enrichment analyses (Figure S1) on the UpRGs and DownRGs. Interestingly, we found that the UpRGs were enriched with the pathways that include the regulation of insulin-like growth factor receptor signaling and neuroblast proliferation, while the DonwRGs were enriched with the pathways that include neuron remodeling and the regulation of synapse organization. The results of the GO pathway analysis indicate that neurodevelopment might be affected by differentially expressed genes that function in neuron proliferation, apoptosis, remodeling, and assembly. The KEGG and Wiki pathway analyses further identified the pathways associated with circadian rhythms, neural crest differentiation, and the Prader–Willi and Angleman syndromes, which are genetic disorders with high comorbidities with ASD (Figure S1). We further inferred the protein–protein interaction (PPI) networks of UpRGs and DownRGs through the STRING database. Two PPI networks were constructed with the following properties: the UpRGs PPI network had 202 nodes and 729 edges, with an average node degree of 7.29; and the DownRGs PPI network had 82 nodes and 140 edges, with an average node degree of 3.41 (Figure 2D). In the UpRGs PPI network, we identified seven known autism risk genes (Simons Foundation Autism Research Initiative, SFARI Gene): PON1, MAGEL2, PPP1R1B, SLC29A4, TTC25, DYDC2, and FAM92B that played the central role in the clustered PPI network (red circle in Figure 2D); and six known autism risk genes: TTN, TCF7L2, RORA, FOXP2, RIMS3, and SATB2 in the DownRGs PPI network (green circle in Figure 2D). By qRT-PCR, we validated that Pon1, Megal2, Dydc2, Fam92b, and Tcf7l2 were indeed differentially expressed in the hippocampi of SD mice (Figure 2E).

**Figure 3:**
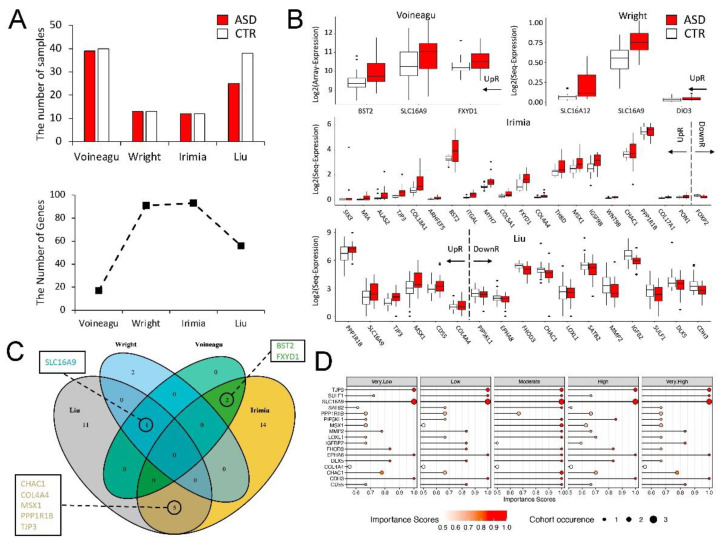
**Multiple DEGs in the hippocampi of SD mice are identified as ASD risk genes in four published studies.** (**A**) Top panel bar plot shows the number of ASD and CTR samples in each of the four published cohorts; bottom panel line plot shows the number of mouse-model-identified DEGs that also measured the expression in the cohort. (**B**) Boxplots show the expression values of overlapped DEGs between mouse models and each cohort (mice-cohort DEGs). There are 3, 3, 21, and 17 SD mice DEGs that are also differentially expressed in the cohort data of Voineagu et al., Wright et al., Irimia et al., and Liu et al., respectively. The genes in the boxplot are ordered by the Log2FC values. (**C**) Venn diagram shows the overlapping genes between mouse-cohort DEGs. We highlight that SLC16A9 is identified in three cohorts, and that CHAC1, COL4A4, MSX1, PPP1R1B, TJP3, BST2, FXYD1 are identified in two cohorts. (**D**) Dot plots show the importance score of genes for each ADI-R class in the data of Liu et al. The importance scores are calculated from the learning vector quantization model and are characterized as: Very Low, Low, Moderate, High, Very High, each corresponding to the low, moderate2, moderate1, high2, and high1 ADI-R classes in the original paper, respectively. The larger the importance score, the more the importance of the gene to predicting the ADI-R class. The size of the dot represents the occurrence of the gene in different cohorts.

We further examined DEGs identified in SD mice to search for the ASD-associated genes in four public human ASD cohorts, with a total of 89 ASD and 103 CTR transcriptome data. The ratios of the ASD/CTR samples are: 39/40 for the Voineagu cohort; 13/13 for the Wright cohort; 12/12 for the Irimia cohort; and 25/38 for the Liu cohort (Figure 3A). We were able to overlap the 17, 91, 93, and 56 SD DEGs in the Voineagu, Wright, Irimia, and Liu cohorts, respectively (Figure 3A). We then applied a *t*-test with the linear regression model on each overlapped gene, and found that the 3, 3, 21, 17 SD DEGs were also differentially expressed in the Voineagu, Wright, Irimia, and Liu’s cohorts, respectively, and, thus, we denoted them as mouse-cohort DEGs (Figure 3B and Figure S3). Intriguingly, eight of these genes were shared by at least two cohorts (Figure 3C). In particular, SLC16A9 was found to be significantly upregulated in three cohorts, indicating that it might be used as a potential biomarker or therapeutic target. Next, we used the learning vector quantization algorithm to calculate the importance scores of the 17 genes identified in the Liu cohort on the basis of the Autism Diagnostic Interview-Revised (ADI-R) class annotation of patients (Figure 3D). We found that SLC16A9, TJP3, CDH3, and EPHA8 had the highest importance scores for all ADI-R classes, implying that these four genes may be used to predict ASD. Taken together, these results indicate significant overlaps of the SD DEGs with SFARI genes and autism-cohort DEGs.

### 3.3. Hyperactivation of mTOR and ERK MAPK Pathways in the Hippocampi of SD Mice

**Figure 4:**
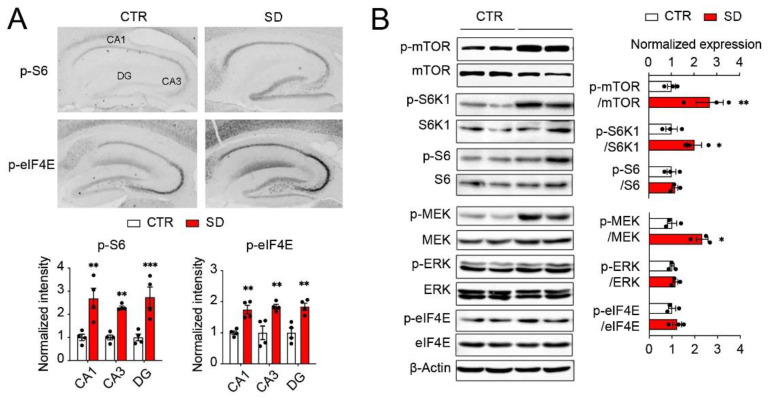
**Hyperactivation of mTOR and ERK MAPK pathways in the hippocampi of SD mice.** (**A**) Representative microscopic images of the sagittal hippocampus sections immunostained for p-S6 and p-eIF4E in the CTR and SD mice. Quantitation of staining intensities in hippocampal CA1, CA3, and DG areas are shown below. Note significant increases in the p-S6 and p-eIF4E levels in the hippocampi of SD mice. Data are displayed as individual values and mean ± SEM. n = 4 in CTR and SD. *** *p* < 0.001; ** *p* < 0.01 vs. CTR. (**B**) Western blots indicate upregulation of mTOR/S6K1/S6 and MEK/ERK/eIF4E kinase activities in the forebrains of SD mice compared with CTR mice. Quantitative analysis is shown to the right. Data are displayed as individual values and mean ± SEM. n = 3 in CTR and SD. ** *p* < 0.01; * *p* < 0.05 vs. CTR.

Our previous work indicates that the activities of the mTOR and MAPK pathways are regulated by the circadian clock. As insulin and the TLR4-associated signaling pathways were strongly represented in the DEGs in the SD hippocampi (Figure 2C), we next determined whether the activities of the mTOR and MAPK pathways were changed in the SD hippocampi because of circadian disruption. We first examined the levels of p-S6, a marker of the mTOR pathway activities, and the level of p-eIF4E, a marker of the MAPK/MNK pathway, in the sagittal brain sections by immunostaining. Interestingly, we found the upregulation of the levels of p-S6 and p-eIF4E in multiple brain regions, including the hippocampus. Quantitative analysis revealed significantly increased levels of p-S6 and p-eIF4E in the CA1, CA3, and DG of the hippocampi in the SD mice compared to the CTR mice (Figure 4A, p-S6: *p* < 0.0001; p-eIF4E: *p* < 0.0001). To further confirm the immunostaining data, we examined the activities of the mTOR/S6K1/S6 and MEK/ERK/eIF4E pathways in the forebrains by Western blotting. Indeed, we found the significant upregulation of the p-mTOR, p-S6K1, and p-MEK levels in the forebrains of SD mice compared with CTR mice. However, we did not detect a significant upregulation of the p-S6 and p-eIF4E levels by Western blotting. The discrepancy between immunostaining and Western blotting may be due to the different brain regions (hippocampus vs whole forebrain) used for analysis. Taken together, these results indicate the hyperactivities of the mTOR/S6K1 and ERK/MAPK pathways (Figure 4B).

### 3.4. Aberrant Synaptic Transmission, Dendritic Spine Morphology, and Protein Synthesis in the Hippocampi of SD Mice

**Figure 5:**
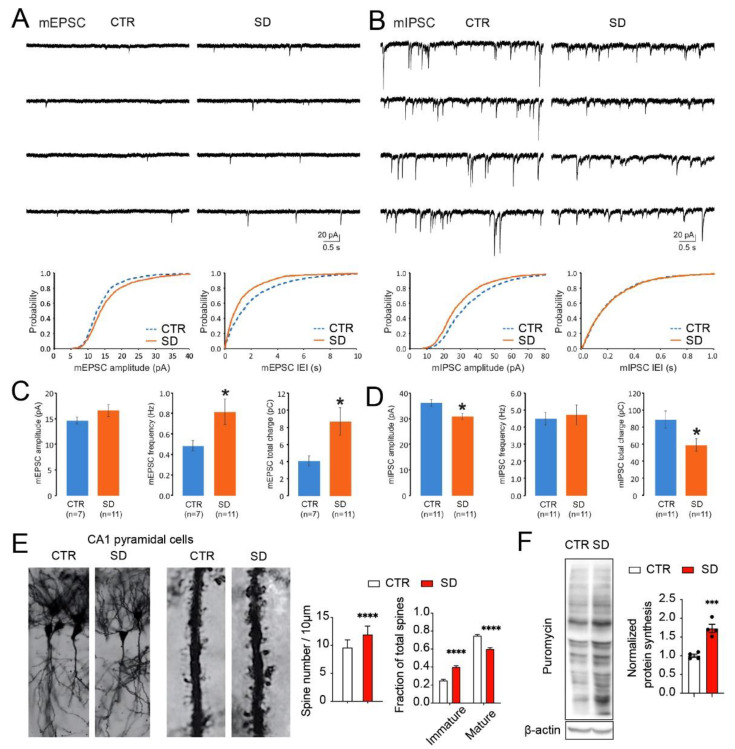
**Aberrant synaptic transmission, dendritic spine morphology, and protein synthesis in the hippocampi of SD mice.** (**A**) Representative traces and cumulative probabilities (amplitude and inter event intervals, IEIs) of mEPSC recordings from hippocampal CA1 neurons. Quantitative analysis is shown below and in (**C**). * *p* < 0.05 vs. CTR. Note that the frequency and total charge, but not the average amplitude, were increased in the hippocampal CA1 neurons of SD mice compared with CTR mice. (**B**) Representative traces and cumulative probabilities (amplitude and inter event intervals, IEIs) of mIPSC recordings from hippocampal CA1 neurons. Quantitative analysis is shown below and in (**D**). * *p* < 0.05 vs. CTR. Note that the amplitude and total charge, but not the frequency, were decreased in the hippocampal CA1 neurons of SD mice compared with CTR mice. The frequency is the reciprocal of IEI. The total charge is the summation of the area integrals of mEPSCs (in a 3-min recording period) or mIPSCs (in a 1-min recording period). n denotes the number of neurons from >3 mice for each group. (**E**) Representative microscopic images of CA1 pyramidal neurons and dendritic spines afterGolgi–Cox staining. Quantitative analysis indicates that spine density and the fraction of immature dendritic spines were increased in dendrites of hippocampal CA1 neurons in SD mice compared with the CTR mice. Data are displayed as mean ± SEM. **** *p* < 0.0001; n = 51 neurons from 4 mice for CTR and SD groups. (**F**) A de novo protein synthesis assay by puromycin incorporation revealed the higher rate of global protein synthesis in the forebrains of SD mice compared with CTR mice. *** *p* < 0.001, n = 4 for CTR and SD mice.

As DEGs that are related to synaptic organization, axon guidance, and neural development were identified, we next investigated the potential changes in the synaptic functions in the hippocampi of SD mice. By electrophysiological recording from the CA1 pyramidal neurons, we measured miniature excitatory postsynaptic currents (mEPSCs) and miniature inhibitory postsynaptic currents (mIPSCs) after blocking the GABA receptor (with bicuculline) and the glutamate receptors (with NBQX and APV), respectively. Compared to the control group, we found that the frequency and total charge of the mEPSCs were significantly increased (*p* < 0.05; Student’s *t*-test) while the amplitude and total charge of the mIPSCs were reduced substantially (*p* < 0.05; Student’s *t*-test) (Figure 5A–D). These results indicate that circadian disruption led to an imbalance of the excitatory and inhibitory inputs in the hippocampal circuits of SD mice.

Next, we studied the morphology of dendritic spines in the CA1 pyramidal neurons by Golgi–Cox staining. We found a significant increase in the total spine density of the distal dendrites in the hippocampus, CA1 (Figure 5E *p* < 0.0001; Student’s *t*-test), with a corresponding decrease in the mature fraction, and an increase in the immature fraction of total spines (*p* < 0.0001; two-way ANOVA). These results demonstrate the immature morphology of dendritic spines. The imbalance of excitatory and inhibitory inputs and immature spine morphology together indicate neurodevelopmental deficits in the SD mice, consistent with the findings by RNA-seq. Lastly, to study global protein synthesis in the SD brains, we used puromycin incorporation to label newly synthesized proteins. Strikingly, we found an ~2-fold increase in the global protein synthesis rates in the brains of the SD mice (Figure 5F, *p* = 0.007; Student’s *t*-test), which indicates dysregulated translational control and is consistent with the hyperactivation of the translational control pathways (mTOR and MAPK pathways) (Figure 4). Together, these results demonstrate the consistent cellular and molecular changes in the hippocampi of SD mice.

### 3.5. Autistic-like Behavioral Changes in SD Mice

**Figure 6:**
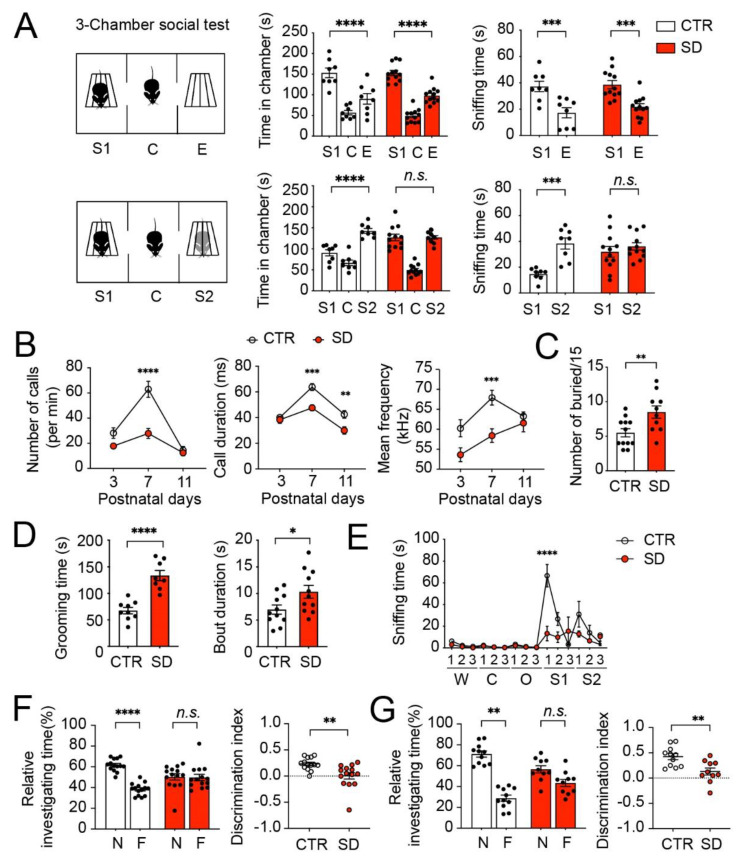
**Autistic-like behavioral changes in SD mice.** (**A**) Three-chamber test for mouse sociability. Left: a schematic diagram. S1: stranger 1; C: center; E: empty; S2: stranger 2. Right: bar graphs indicating time spent in individual chambers and time spent sniffing wire cages during the three-chamber test. Note that both CTR and SD mice spent longer times in the S1 chamber and sniffing the S1 cage, compared with in the E chamber and sniffing the E cage. CTR mice spent longer times in the S2 chamber and sniffing the S2 cage, compared with in the S1 chamber and sniffing the S1 cage. In contrast, SD mice spent similar times in the S1 and S2 chamber and sniffing the S1 and S2 cage. Data are displayed as individual values and mean ± SEM. *** *p* < 0.001; **** *p* < 0.0001; n.s., not significant. Data are displayed as individual values and mean ± SEM. n = 8 in CTR and n = 12 in SD. (**B**) Ultrasonic vocalization tests at Postnatal Day (P) 3, 7, and 11. Note that SD pups exhibited a reduced number of calls, call durations, and call frequencies at P7 compared with the CTR pups. Data are displayed as mean ± SEM ** *p* < 0.01; *** *p* < 0.001; **** *p* < 0.0001 vs. CTR; n = 5–6 for P3 and P11 in CTR and SD; n = 8-9 for P7 in CTR and SD. (**C**) A bar graph indicates the numbers of marbles buried in the marble burying test. n = 12 in CTR and n = 10 in SD. Data are displayed as individual values and mean ± SEM. ** *p* < 0.01. (**D**) Spontaneous self-grooming test. Note that SD (n = 8) mice displayed increased grooming time and duration of bouts as compared with WT (n = 9) mice. * *p* <0.05; **** *p* < 0.0001 vs. CTR (**E**) Olfactory habituation test. W: water; C: cinnamon; O: food (butter); S1: social odor 1 (dirty cage swab1); S2: social odor 2 (dirty cage swab2). Note that time spent sniffing the social odor was markedly reduced, whereas time sniffing nonsocial odors was not changed in the SD mice compared with the CTR mice. **** *p* < 0.0001 vs. CTR. n = 4 mice in CTR and SD. Data are presented as mean ± SEM. (**F**) Novel object recognition test. During the training session, mice were exposed to two familiar (F) objects. After 24 h, one F objectwas replaced by a novel (N) object in the testing session. Time animals spent investigating the two objects was measured. The discrimination index was calculated as Time_novel_ − Time_familiar_/Time_novel_ + Time_familiar_. CTR (n = 14) mice spent a longer time investigating the N object compared with the F object, whereas the SD (n = 14) mice spent similar time investigating the N and familiar objects. The discrimination index was decreased in the SD mice compared with the CTR mice. ** *p* < 0.01; **** *p* < 0.0001 vs. CTR; n.s., not significant. (**G**) Object location memory test. During the training session, mice were exposed to two identical objects. After 24 h, one object was left at the same familiar (F) location and the other object was moved to a novel(N) location. Time animals spent investigating the two objects was measured. The discrimination index was calculated as Time_novel_ − Time_familiar_/Time_novel_ + Time_familiar_. CTR (n = 10) mice spent a longer time investigating the object in the Nlocationas compared with the object in the Flocation, whereas SD (n = 11) mice spent similar time investigating the objects in the Nlocation compared with the object in the Flocation. The discrimination index was decreased in the SD mice compared with the CTR mice. Data are displayed as individual values and mean ± SEM. ** *p* < 0.01; n.s., not significant.

Hippocampal RNA-seq identified DEGs in the SD mice that are associated with the Prader–Willi and Angelman syndromes, two genetic disorders associated with ASD, and we identified significant overlaps between SD DEGs and human autism risk genes (Figure 3). The genetic, cellular, and molecular changes described above suggest autistic-like phenotypes in SD mice. These results led us to further investigate behavioral changes in SD mice using a battery of behavioral tests. We first performed the three-chamber test to evaluate mouse social behavior. In the sociability test, similar to the CTR mice, the SD mice exhibited a preference for the stranger mouse (S1) compared with the empty cup (E), as was indicated by the mouse spending more time in the chamber with S1 than in the chamber with E, and the more time spent sniffing the S1 cage than the E cage (Figure 6A, two-way ANOVA, chamber x group interaction, *p* = 0.603; sniffing time, x group interaction, *p* = 0.628). These results indicate the intact sociability in the SD mice. In contrast to the CTR mice, however, the SD mice displayed no preference for a novel stranger mouse (S2) compared with the familiar mouse (S1), as indicated by the similar time spent in the chamber with S1 and S2, and similar time spent sniffing S1 and S2 (Figure 6A, two-way ANOVA, chamber x group interaction, *p* < 0.0001; sniffing time, x group interaction, *p* = 0.011). These results indicate an impaired preference for social novelty in SD mice.

Next, we used the USV test to investigate social communication via the vocalization between pups and the mother. The SD pups displayed fewer calls and lower call frequencies at postnatal day 7 (P7), and shorter call durations at P7 and P11 compared with the CTR pups, indicating impaired social communication in the SD pups (Figure 6B, two-way ANOVA, number of calls, age x group interaction, *p* = 0.003; call duration, age x group interaction, *p* = 0.020; mean frequency group, *p* = 0.0007, age x group interaction, *p* = 0.130). Repetitive behaviors are also core symptomatic traits in ASD. We assessed mouse repetitive behaviors by marble burying and grooming. In the marble burying test, the SD mice buried ~2-fold more marbles compared with the CTR mice (Figure 6C, Student’s *t*-test, *p* = 0.0095). The SD mice also exhibited increased total grooming times and longer bout durations compared with the CTR mice in a spontaneous self-grooming test (Figure 6d, grooming time, Student’s *t*-test, *p* < 0.0001; bout duration, Student’s *t*-test, *p* = 0.018). These results indicate that the SD mice exhibited excessive repetitive behaviors. Next, we used an olfactory habituation/dishabituation test to assess whether SD mice can detect and differentiate nonsocial odors from social odors. Interestingly, the SD mice spent decreased amounts of time investigating the social odors compared with CTR mice, whereas there was no difference in the times spent investigating nonsocial odors between the SD and CTR mice, which indicates that olfactory processing remained intact, but olfactory social preference was impaired in the SD mice (Figure 6E, two-way ANOVA, odor x group interaction, *p* < 0.0001).

Lastly, we investigated the potential changes in the cognitive functions in the SD mice using the novel object recognition (NOR) and object location memory (OLM) tasks, both of which are dependent on the hippocampus. As shown in Figure 6F, 24 h after acquisition, the CTR mice, but not the SD mice, exhibited a preference for the novel object over the familiar object in the NOR task (Figure 6F, two-way ANOVA, object x group, *p* < 0.0001). Similarly, in the OLM tasks, the CTR mice, but not the SD mice, exhibited a preference for the object at a novel location over the object at a familiar location (Figure 6G, two-way ANOVA, location x group, *p* < 0.0001). The discrimination indexes (DI) of the SD mice were significantly decreased in both tasks in the SD mice compared with the CTR mice (Figure 6F–G, NOR DI: Student’s *t*-test, *p* = 0.003; OLM DI: Student’s *t*-test, *p* = 0.004). These results indicate that the long-term spatial memory was impaired in SD mice. Taken together, these behavioral changes collectively indicate autistic-like behavioral changes in the SD mice, which are consistent with the molecular, cellular, and genetic changes identified in the brains of these mice.

## 4. Discussion

Circadian dysfunction has been increasingly linked to ASD by clinical evidence, but experimental evidence is still needed in order to establish a causal relationship between circadian rhythm disruption and autistic-like phenotypes. Animal models are commonly used to investigate the causal roles of the genetic and environmental factors in ASD etiology. Here, taking advantage of a novel SD mouse model, we found that the disruption of the circadian rhythms impairs neurodevelopment and leads to autistic-like behavioral phenotypes in adulthood. Using a multidisciplinary approach, we also elucidated the genetic, molecular, and cellular mechanisms underlying these phenotypes. Together, our results demonstrate the significant role of circadian rhythms in neurodevelopment and provide the first experimental evidence that links environmental circadian disruption to the pathogenesis of ASDs.

Our transcriptomic analysis identified several interesting pathways that are related to neurodevelopment, such as neuronal proliferation, remodeling, and synaptic organization, indicating that the dysregulation of multiple genes involved in neurodevelopment may play an important role in driving the autistic-like phenotype in SD mice. The RNA-seq data demonstrate genetic similarities between our SD model and human autism. We were able to identify a set of 35 mouse-cohort DEGs. In particular, SLC16A9 was not only found in three of the four cohorts, but also has the highest importance score for all ADI-R classes, implying that it may underlie the phenotypes of SD mice and can potentially be used to predict ASD in human patients.

We focused on the hippocampus for molecular and cellular analysis, as it is important for cognitive functions and has been implicated in autism pathogenesis. It is also one of the extra-SCN regions exerting robust circadian oscillations [35,36]. Previous studies have revealed the dependence of circadian rhythms on learning and memory in animals [35,37], especially in hippocampus-dependent long-term spatial memory. Indeed, we found that the molecular circadian oscillations were disrupted in the SD mice. As a result, the long-term spatial memory was impaired, as demonstrated by the NOR and OLM tests. Moreover, the disruption of the excitation–inhibition (E/I) balance is considered a hallmark in the ASD mouse models [27]. We performed mEPSC and mIPSC recordings in hippocampus CA1 pyramidal neurons and found an increased total charge of mEPSCs, but a decreased total charge of mIPSCs. The E/I imbalance in the hippocampus may also account for the behavioral changes in the SD mice.

The circadian clock and autism are linked by common translational control pathways in the brain. Our work found that the mTOR (mammalian target of rapamycin)/eIF4E (eukaryotic translation initiation factor 4E) axis regulates fundamental functions of the circadian clock [14,38,39]. Interestingly, the mTOR pathway is frequently dysregulated in developmental brain malformations, including autism, and is pharmacologically targeted to treat certain types of autism [40,41]. Dysregulation of the mTOR/eIF4E axis disrupts the clock and engenders ASD-like phenotypes in animals, indicating potential crosstalk between the brain clock and autism. Since aberrant synaptic morphologies were observed in some ASD-models, such as Fragile X syndrome [42,43,44] and Rett syndrome [45,46], it was suggested as one of the developmental factors affecting synaptic transmission and activity-dependent synaptic plasticity. PTEN and TSC1, as mTOR repressors, were proven to be related to dendritic and spine morphologies [47,48,49]. We found hyperactivation of the mTOR and MAPK pathways in SD brains, which was accompanied by aberrantly immature dendritic spine morphologies and increased protein synthesis. Dysregulated translational control may be the biochemical basis underlying the functional changes in the SD mice.

## 5. Conclusions

Using a combination of genetic, electrophysiological, biochemical, and behavioral approaches, our study demonstrates the fundamental significance of circadian rhythms in neurodevelopment and provides experimental evidence that links environmental circadian disruption to the pathogenesis of autism spectrum disorders. The identified molecular mechanisms that link circadian dysfunction to autism (mTOR, MAPK, autism risk genes, etc.) could transform our understanding of ASD pathogenesis and provide new hope for ASD patients. The SD mouse is a new mouse model that links circadian disruption to ASD-like phenotypes. It provides a preclinical tool for investigating the causal role of genetic mutations and environmental factors in the etiology of ASD, and for performing the preclinical screening of pharmacological agents.

## Data Availability

The hippocampal RNA-seq data that support the findings of this study are available at GEO repository, GSE189289.

## Supplementary Materials

The following are available online at https://www.mdpi.com/article/10.3390/cells10123314/s1: Figure S1: Top pathways categories of DEGs as identified by KEGG and Wiki pathway analysis; Figure S2: Linear regression model identified mouse-cohort DEGs; Table S1: A complete list of DEGs in the hippocampi of SD mice.
