# Supplementary figures and images for: Disruption of Circadian Rhythms by Ambient Light during Neurodevelopment Leads to Autistic-like Molecular and Behavioral Alterations in Adult Mice
Source: Cells. 2021 Nov 26;10(12):3314. doi: 10.3390/cells10123314 (PMC8699695; doi:10.3390/cells10123314)

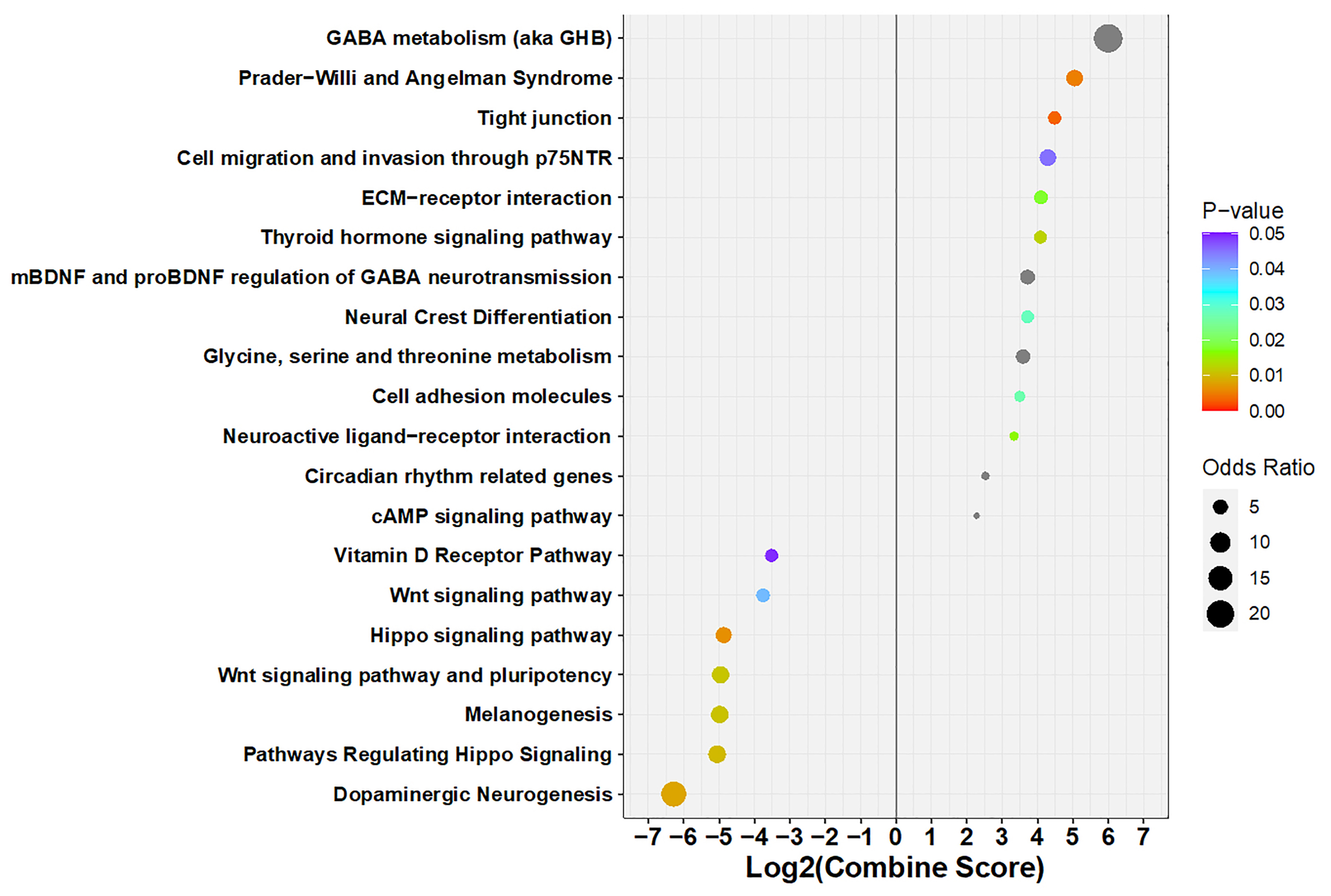

Supplement: Supplementary file 1 [file cells-10-03314-s001.zip › cells-1481445-sm/Figure S1.jpg]

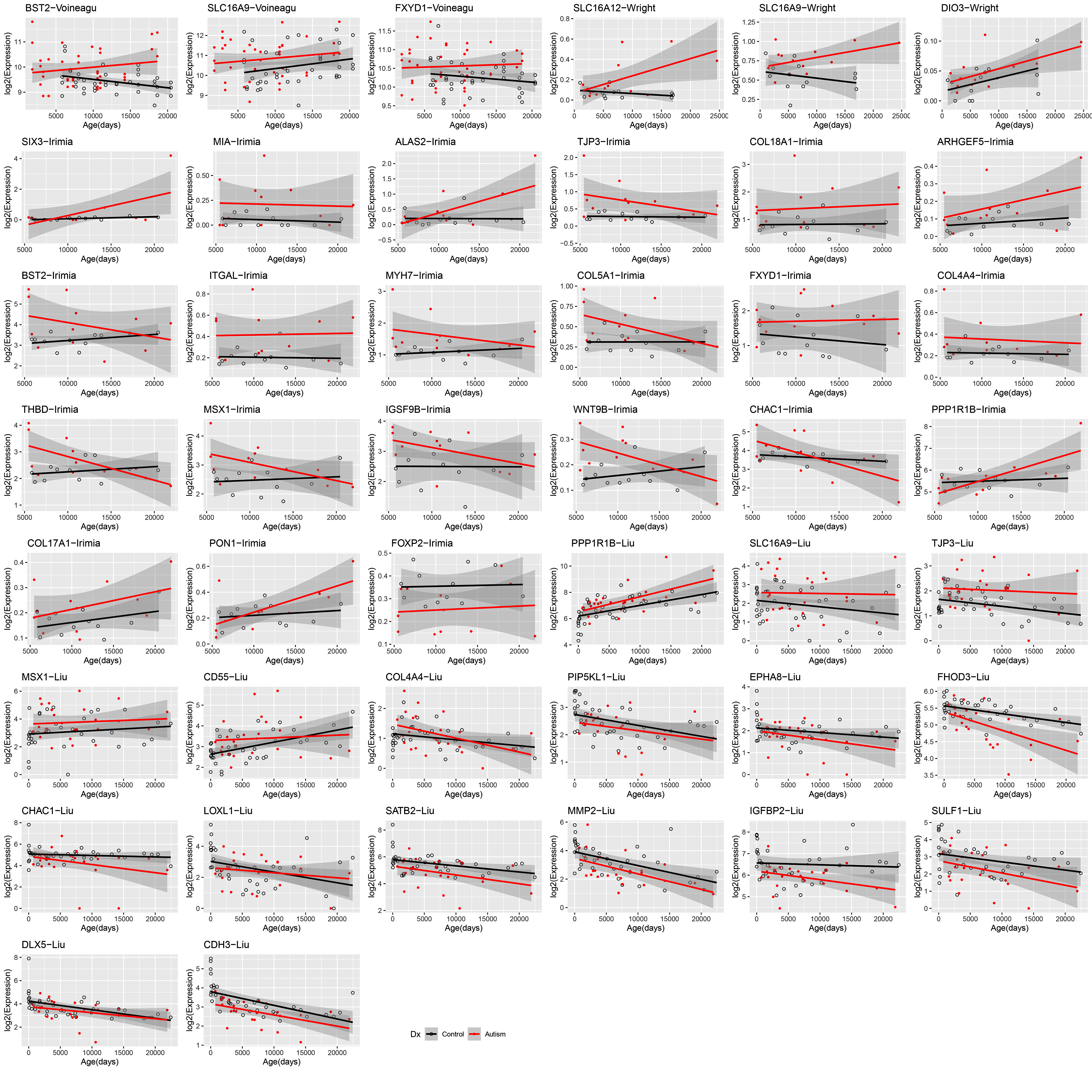

Supplement: Supplementary file 1 [file cells-10-03314-s001.zip › cells-1481445-sm/Figure S2.jpg]
